# Supplementary material for: UNILIPID, a Methodology for Energetically Accurate Prediction of Protein Insertion into Implicit Membranes of Arbitrary Shape
Source: Membranes (Basel). 2023 Mar 21;13(3):362. doi: 10.3390/membranes13030362 (PMC10054542; doi:10.3390/membranes13030362)
Supplement: Supplementary file 1 [file membranes-13-00362-s001.zip › membranes-2240102-supplementary.pdf]

Supplementary Information

UNILIPID, a Methodology for Energetically Accurate Prediction of Protein Insertion into Implicit Membranes of Arbitrary Shape

André Lanrezac and Marc Baaden *

Université Paris Cité, CNRS, Laboratoire de Biochimie Théorique, 13 rue Pierre et Marie, Curie, F-75005 Paris, France
* Correspondence: baaden@smplinux.de; Tel.: +33-1-58-41-51-76

Abstract: The insertion of proteins into membranes is crucial for understanding their function in many bio-logical processes. In this work, we present UNILIPID, a universal implicit lipid-protein descrip-tion as a methodology for dealing with implicit membranes. UNILIPID is independent of the scale of representation and can be applied at the level of all atoms, coarse-grained particles down to the level of a single bead per amino acid. We provide example implementations for these scales and demonstrate the versatility of our approach by accurately reflecting the free energy of transfer for each amino acid. In addition to single membranes, we describe the analytical implementation of double membranes and show that UNILIPID is well suited for modeling at multiple scales. We generalize to membranes of arbitrary shape. With UNILIPID, we provide a methodological framework for a simple and general parameterization tuned to reproduce a selected reference hydrophobicity scale. The software we provide along with the methodological description is op-timized for specific user features such as real-time response, live visual analysis, and virtual real-ity experiences.

Keywords: implicit membrane; lipid bilayer insertion; hydrophobicity scale; coarse-grained representation

S2. Materials and Methods

2.1.1. Atom Type Calibration Method 1

In this approach, we aim to keep the proportions of the energies between the 7 atom types. A reference type is chosen, we settled on the carbon Csp3, and for all other atom types the ratio to the reference value of Csp3 (−0.1050 kJ.mol^{-1} Å^{-2}) is determined. The result is shown in Table S1.

Table S1. Transfer energy[1] ratio with respect to reference atom type Csp3.

Atom Types	Original E_{tr} kJ mol^{-1} Å^{-2}	Ratio[2]
Csp3	−0.1050	1.000000
Csp2	−0.0134	0.127619
Hnc	−0.0397	0.378095
Hc	0.0362	−0.344762
O	0.0403	−0.383810
N	0.1120	−1.066667
S	−0.1080	1.028571

[1] Data taken from [26]. [2] Ratio defined as $E_{tr/type}$ / $E_{tr/Csp3}$

The E_{tr} values for each atom type are adjusted as follows:

$$E_{tr}(n) = E_{tr} + \Delta E_{tr}(n) \times \text{Ratio}, \tag{S1}$$

$$\Delta E_{tr}(n) = \Delta E_{tr}(n\text{-}1) + \text{sign}(n) \times \delta E_{tr}, \tag{S2}$$

$$\text{sign}(n) = -1 \times \text{sign}(n\text{-}1) \text{ if } | E_{IMP}(n) - E_{tr/EXP} | > | E_{int}(n\text{-}1) - E_{tr/EXP} |, \text{ else sign}(n) = 1 \tag{S3}$$

In other words, the adjusted transfer energies with constant relative ratio are calculated by adding a small energy variation whose sign changes as the total energy of the side-chain atoms at iteration n moves away from the experimental energy compared with step n−1. In this way, E_{IMP} converges to $E_{tr/EXP}$ at each step and the algorithm is stopped when the difference between the two values is less than a certain arbitrary value, which in our implementation is 0.1 kJ mol^{-1} by default. However, keeping the ratios between the parameters constant may mean that the individual values differ greatly from the initial reference values in order to obtain a total side-chain transfer energy that is close to the experimental value.

2.1.2. Atom Type Calibration Method 2

In this variant of method 1, strictly the same $\Delta E_{tr}(n)$ value is applied to each of the atom types, hence the ratio is no longer needed. In this case equation (3) becomes:

$$E_{tr}(n) = E_{tr} + \Delta E_{tr}(n), \tag{S4}$$

This method does not preserve the ratio between the relative contributions of each atomic type parameter, but in practice this procedure leads to less significant adjustments in their absolute values.

2.1.3. Atom Type Calibration Method 3

This method is a variant of method 2, with the additional condition that for each atom type the sign of $E_{tr}(n)$ matches the sign of the initial reference transfer energy of the given type. Thus, reference parameters of opposite sign will have variations $\Delta E_{tr}(n)$ that are also of opposite sign. The variations are bound to a limiting factor (in this case a sigmoid function) to avoid sign changes for parameters approaching 0. This method preserves a certain physicochemical signature of the atom types.

2.1.4. Atom Type Calibration Method 4

This method was developed by trial and error to reduce some shortcomings of the previous three methods in fitting the parameters. Given a total of N iterations, the difference in applied energy depends on the iteration, i.e., it increases at each stage, as does the acceptance condition that terminates the iterative procedure. This termination condition for methods 1 to 3 was that the difference in the total transfer energy calculated at step n compared to that of the experiment is less than 0.1 kJ mol^{-1}. For method 4, this condition is defined as n / N, so we theoretically accept a maximum difference of 1.0 kJ mol^{-1}, but this never occurs in practice. For some amino acids, such as Phe, where it is difficult to converge to target values, a slightly higher difference from the experimental reference value can be accepted using this approach. The energy difference applied to each parameter, which depends on n, is inversely proportional to the relative deviation of the parameter from its reference value. Thus, the variation in the energy applied to each parameter decreases as one moves away from its reference value. The goal is to vary the parameters by specific adjustments according to their sign as in method 3 and to weight them heavily to limit the deviations. The method is presented as pseudo code in Figure 1.

```
N = 100000 // set number of iterations
sign = 1 // initialize signed adjustment variable
ind = 0 // initialize iteration adjustment variable, depend-
ent on the sign variable
reference_parameters = get_initial_parameters() // retrieve
starting parameter set
current_parameters = reference_parameters    // start from
the starting parameter set
E_tr_exp = get_experimental_Etr(“Ala”) // get target en-
ergy for Alanine
E_int_n-1 = 99999 // initialize value for the first pass of the
loop

// main loop
for n = 0 to N-1
    E_int_n = compute_IMPALA_energy(current_parame-
ters)
    delta_E = E_int_n - E_tr_exp    // current difference to
target value

    if abs(delta_E) < n/N then
        break    // we have reached desired accuracy and
exit refinement
    end if

    if abs(delta_E) > abs(E_int_n-1 – E_tr_exp) then
        sign =* -1   // change sign if we did not make pro-
gress compared to previous iteration
    end if

    ind += sign × i
    error_parameters = current_parameters/reference_pa-
rameters - 1
    delta_parameters = (ind/N) * 1/(1- error_parameters)
    current_parameters = reference_parameters +
delta_parameters

    E_int_n-1 = E_int_n    // memorize current E_int_n
value for next step
end for

// current_parameters now contains the refined parameter
set for Alanine
```

Figure S1. Pseudo-code for calibration method 4 applied to a given amino acid, here alanine.

S3. Results

S3.1. Comparison of Previous IMPALA Implementation with UNILIPID

Table S2. UNILIPID parameter set calibrated to reproduce the hydrophobicity scale of Fauchère and Pliska. Values for the first ten amino acids. [1.]

Type	ALA	ARG	ASN	ASP	CYS	GLN	GLU	GLY	HIS	ILE
Csp3	*-0.0731*	*-0.1008*	*-0.1177*	*-0.0461*	*-0.1405*	*-0.1901*	*-0.0510*	-0.1050	*-0.2990*	*-0.1676*
Csp2	-0.0134	*-0.0133*	*-0.0137*	*-0.0079*	-0.0134	*-0.0141*	*-0.0089*	-0.0134	*-0.0142*	-0.0134
H(=0)	*-0.0353*	*-0.0394*	*-0.0405*	*-0.0264*	*-0.0412*	*-0.0417*	*-0.0285*	*-0.0258*	*-0.0420*	*-0.0416*
H(/0)	0.0362	*0.0365*	*0.0356*	0.0362	*0.0350*	*0.0346*	0.0362	0.0362	*0.0344*	0.0362
O	0.0403	0.0403	*0.0395*	*0.0721*	0.0403	*0.0384*	*0.0619*	0.0403	0.0403	0.0403
N	0.1120	*0.1188*	*0.1013*	0.1120	0.1120	*0.0904*	0.1120	0.1120	*0.0886*	0.1120
S	-0.1080	-0.1080	-0.1080	-0.1080	*-0.1542*	-0.1080	-0.1080	-0.1080	-0.1080	-0.1080

[1] The values in italics have been adjusted, the values in bold are the values whose signs have changed, while all other values are identical to the reference values.

Table S3. UNILIPID parameter set calibrated to reproduce the hydrophobicity scale of Fauchère and Pliska. Values for the remaining ten amino acids. [1.]

Type	LEU	LYS	MET	PHE	PRO	SER	THR	TRP	TYR	VAL
Csp3	*-0.1676*	*-0.0509*	*-0.1023*	*-0.1594*	*-0.0906*	*-0.0937*	*-0.1027*	*-0.2411*	*-0.6684*	*-0.1339*
Csp2	-0.0134	-0.0134	-0.0134	*-0.0490*	-0.0134	-0.0134	-0.0134	*-0.0518*	*-0.0144*	-0.0134
H(=0)	*-0.0416*	**0.0144**	*-0.0395*	*-0.1276*	*-0.0383*	*-0.0387*	*-0.0395*	*-0.1464*	*-0.0427*	*-0.0410*
H(/0)	0.0362	*0.0903*	0.0362	0.0362	0.0362	*0.0371*	*0.0364*	**-0.0493**	*0.0339*	0.0362
O	0.0403	0.0403	0.0403	0.0403	0.0403	*0.0414*	*0.0405*	0.0403	*0.0376*	0.0403
N	0.1120	*0.1661*	0.1120	0.1120	0.1120	0.1120	0.1120	0.1120	0.1120	0.1120
S	-0.1080	-0.1080	*-0.1050*	-0.1080	-0.1080	-0.1080	-0.1080	-0.1080	-0.1080	-0.1080

[1] The values in italics have been adjusted, the values in bold are the values whose signs have changed, while all other values are identical to the reference values.

3.3. Effect of Mesh Parameters on the Quality of Results

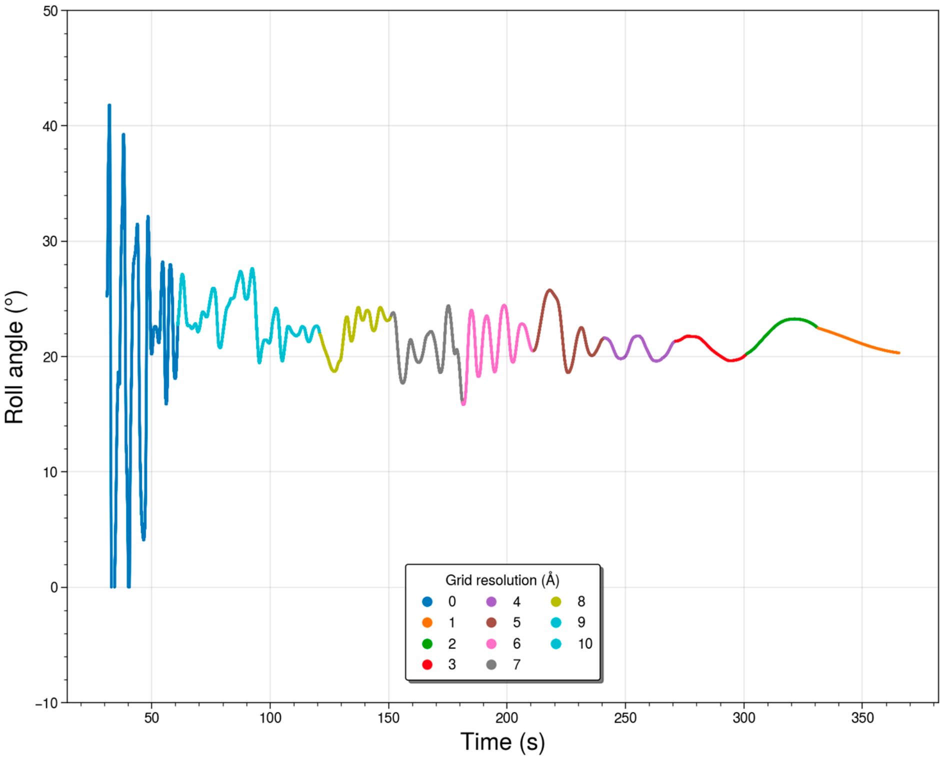

Figure S2. Interactive insertion of OmpA with IMPALA and the UNILIPID flat membrane mesh at different grid resolutions compared to an analytical membrane representation, given in the legend as "REF". The mesh representation varies lattice spacing with decreasing lattice roughness through a sequence from 10Å to 1Å by 1Å steps. Results are shown for the roll angle. To uniquely define the

roll angle without introducing new reference points, we measure the rotation of the barycenter of the protein about the insertion vector described earlier. We define the zero point when the z-coordinate of the barycenter is lowest during a complete rotation. The time axis on the abscissa is the actual measured time.
